# 45419 Patient Perceptions of COVID-19 Impact on their Fertility Care

**DOI:** 10.1017/cts.2021.621

**Published:** 2021-03-31

**Authors:** Karen Dsouza, Minerva Orellana, Alessandra Ainsowrth, Kirsten A. Riggan, Chandra Shenoy, Megan A. Allyse

**Affiliations:** Mayo Clinic

## Abstract

ABSTRACT IMPACT: In alignment with principles of community engaged research, feedback from participants in this research study may influence infertility clinics to offer psychological support for individuals or couples experiencing high levels of psychosocial burden when pursuing fertility procedures, especially during periods of interrupted care or uncertainty. OBJECTIVES/GOALS: 1) To assess the psychosocial impact on patients whose fertility treatments were interrupted during the COVID-19 shutdown. 2) To assess the correlation of patient perceived risk at the time of treatment cessation compared to the resumption of treatment, both during an ongoing pandemic. METHODS/STUDY POPULATION: Female patients with scheduled fertility treatments at Mayo Clinic within 6 months of 3/15/2020, were contacted through the patient portal and invited to participate in this study. Interested patients were contacted by a study staff member to obtain their consent and HIPAA authorization and to schedule a phone or Zoom interview. Semi-structured interviews were conducted individually, or in partner dyads depending upon participant preference, and were recorded with their permission. Audio recordings were professionally transcribed and de-identified. Transcripts were qualitatively analyzed using NVivo 12 based on the principles of grounded theory. RESULTS/ANTICIPATED RESULTS: 26 participants were interviewed; 20 interviews were conducted individually and 3 were conducted in dyads with their partners. Initial themes from the interviews show that COVID-19 compounded existing psychosocial burden on individuals and couples undergoing fertility treatments. Women who were older in age, had prolonged history of infertility, or multiple unsuccessful treatment cycles reported feeling an increased urgency to proceed with fertility treatments due to the time sensitive care, which outweighed the perceived risks of COVID-19 to either themselves or the potential pregnancy. Patients also reported a desire for improved communication regarding their procedures and overall well-being, as well as options for counseling services for individuals or couples undergoing fertility treatments. DISCUSSION/SIGNIFICANCE OF FINDINGS: Participants indicated the need for increased psychological support for patients pursuing fertility treatments, especially during periods of interrupted care or uncertainty, as highlighted during the COVID 19 pandemic. Offering counseling as a routine part of the treatment process may mitigate this burden.

